# Study on physiological indicators and spectral response characteristics of alfalfa under simulated spontaneous combustion of coal gangue dumps

**DOI:** 10.3389/fpls.2026.1745759

**Published:** 2026-02-04

**Authors:** Meichen He, He Ren, Yanling Zhao, Tingting He, Chunfang Chen, Lifan Zhang, Yanjie Tang

**Affiliations:** 1 College of Geoscience and Surveying Engineering, China University of Mining and Technology, Beijing, China; 2 Academy of Eco-Civilization Development for Jing-Jin-Ji Megalopolis, Tianjin Normal University, Tianjin, China; 3 Advanced Laser Technology Laboratory of Anhui Province, Hefei, China

**Keywords:** alfalfa, coal gangue dumps, heat stress, model prediction, spectral response, spontaneous combustion

## Abstract

**Introduction:**

Coal gangue dumps may still pose a risk of spontaneous combustion even after reclamation, threatening both ecological restoration and surrounding environments. As a dynamic and complex process, vegetation responses to spontaneous combustion heat stress vary across growth stages. Therefore, identifying growth-stage-dependent spectral responses of physiological indicators is critical for the timely and accurate detection of spontaneous combustion in coal gangue dumps.

**Methods:**

In contrast to field-based investigations, this study selected alfalfa (*Medicago sativa* L.), a typical reclaimed herbaceous species on coal gangue dumps, as the study object. Constant high-temperature conditions were established indoors to simulate heat stress associated with coal gangue spontaneous combustion. The spectral response differences of chlorophyll relative content (SPAD) and photosynthetic parameters at different growth stages were analyzed. Correlation analysis combined with the SPA algorithm was applied to identify sensitive physiological indicators, spectral bands, and characteristic parameters related to heat stress. Subsequently, support vector regression (SVR), random forest regression (RFR), and partial least squares regression (PLSR) models were developed (training set: test set = 3:1, n = 60:20) to determine optimal combinations of growth stage, indicators, and spectral features for monitoring spontaneous-combustion-induced heat stress.

**Results:**

(1) during branching, budding, and flowering stages, the SPAD values and photosynthetic parameters of treatment (T) and control (CK) groups exhibited consistent trends; (2) sensitive features included OS2, FDS1, FDS2, TP, NDVI, and FDNDVI; and (3) vegetation indices based on original spectra achieved the highest prediction accuracy, followed by those derived from first-derivative spectra, triangular parameters, first-derivative reflectance, and original spectral bands. Among all growth stages, the budding stage was identified as the optimal observation period, and the SVR model showed the best performance (FDNDVI: R² = 0.77, RMSE = 3.50).

**Discussion:**

This study reveals growth-stage-dependent physiological and spectral responses of vegetation to spontaneous combustion heat stress and provides a theoretical basis and technical reference for ecological monitoring and early warning of spontaneous combustion in reclaimed coal gangue dumps.

## 1 Introduction

Coal gangue, as a primary waste product generated during coal mining and beneficiation processes, is currently one of the largest accumulated solid wastes in China (Abramowicz et al., 2021). Due to its low utilization efficiency, coal gangue are generally abandoned near mining sites, where they accumulate into large heaps (Li and Wang, 2019). Statistics indicate that there are currently over 2,600 large-scale coal gangue dumps in China, with a total accumulated volume exceeding 7 billion tons, occupying approximately 1.5 million km² of land, and increasing at an annual rate of 300 million tons (Song et al., 2022). Certain components of coal gangue, including residual media, pyrite, sulfide minerals, and other organic substances, are highly prone to spontaneous combustion, releasing harmful gases such as CO and SO_2_ as well as inhalable particulate matter into the atmosphere (Li et al., 2021; Li and Wang, 2019; Zhang et al., 2015; He et al., 2025). This poses a serious threat to the natural environment and human health. Furthermore, rainwater erosion can contaminate surrounding soils and groundwater, causing environmental pollution. Studies have shown that even when mining companies implement fire suppression measures and ecological restoration projects on spontaneously combusting coal gangue dumps, the restored dumps still face the risk of re-ignition (Ren et al., 2020, 2023; Ren et al., 2022a). To prevent secondary ecological risks in reclaimed coal gangue dumps, early identification of spontaneous combustion is essential. Through the effective application of ecological restoration technologies and comprehensive management measures, maintaining the evergreen status of coal gangue dumps is of significant practical importance and long-term value for improving regional ecological environments.

Currently, the primary monitoring targets for spontaneous combustion in coal gangue dumps include surface temperature (Anghelescu and Diaconu, 2024; Jiang et al., 2023; Li et al., 2020; Shao et al., 2024; Zhao et al., 2023) and harmful gases (Li et al., 2021; Shao et al., 2023; Zhao et al., 2022). Wang et al. (2020) delineated high-temperature zones by integrating infrared thermal imaging, borehole temperature measurements, and radon concentration data, establishing a risk assessment method for coal gangue spontaneous combustion and analyzing gas toxicity, explosion hazards, and fire trends in construction areas (Wang et al., 2020). Zhao et al. (2022) monitored the temperature of coal gangue stockpiles using heat pipes and monitoring software, developed fitting models for shallow and deep temperatures, and analyzed internal temperature variations (Zhao et al., 2022). Shao et al. (2023) utilized UAV-based infrared oblique photogrammetry combined with custom software to construct 3D temperature models of coal gangue dumps (Shao et al., 2023). Wasilewski (2020) conducted long-term observations of thermal and gaseous activities in coal gangue dumps using thermal imaging cameras and fixed-point boreholes, developed and applied a numerical model based on initial boundary conditions and atmospheric parameters to identify features most likely to influence fire sources in spoil heaps, and proposed a modern method for monitoring fire hazards in coal mine spoil heaps (Wasilewski, 2020).

Vegetation growth is strongly influenced by the environment (Tauqeer et al., 2022; Yang et al., 2022; He et al., 2023), and monitoring vegetation growth status is therefore an effective approach for stress identification. Jin et al. (2023) identified drought stress by monitoring photosynthetic parameters, chlorophyll fluorescence parameters, and proline content in Sargent’s cherry trees (*Prunus sargentii* Rehder) (Jin et al., 2023). Compared with the control group, the growth parameters of lettuce (*Lactuca sativa* L.) seedlings were significantly reduced under drought stress (Shin et al., 2021). Hu et al. (2025) conducted controlled water-stress experiments and found that one-year-old “Suchazao” tea plants exhibited different sensitive physiological indicators under different stress levels, which could serve as effective criteria for identifying varying degrees of water stress (Hu et al., 2025). High-temperature stress induced by spontaneous combustion in coal gangue dumps is the most prominent abiotic stress factor affecting vegetation growth and physiological functioning, and in severe cases it can even lead to open flames at the surface. Studies have shown that under high-temperature stress, plant cells perceive elevated temperatures through the plasma membrane, triggering Ca^2+^ influx and subsequent binding with calmodulin, thereby initiating specific signal transduction pathways and activating heat stress response mechanisms (Wang et al., 2018). The phenotypic effects of high-temperature stress on plants are mainly manifested as leaf yellowing, wilting, and abscission, as well as reduced growth rates and overall dwarfing (Sharma et al., 2020). Meanwhile, high-temperature stress can cause cellular damage and disrupt physiological activities and hormonal regulation (Driedonks et al., 2015). Photosynthesis provides the energy and material basis for plant life activities. Vegetation exchanges CO_2_, water vapor, and other substances with the environment through stomata, and both photosynthesis and stomatal behavior are highly sensitive to environmental fluctuations. High-temperature stress significantly affects photosynthetic processes and stomatal regulation (Sun et al., 2023; Wang et al., 2025; Xu, 1988), while also altering physiological parameters closely associated with photosynthesis, such as the contents of photosynthetic pigments including chlorophyll. Under high-temperature stress, azalea exhibits reduced chlorophyll fluorescence, decreased stomatal characteristics, and a decline in photosynthetic rate (Khan et al., 2025). Vicente et al. reported that temperature variations influence vessel structure, stomatal dynamics, and assimilation capacity of European beech (Fagus sylvatica) in the southern Alps (Vicente et al., 2022). Feng et al. demonstrated that temperature changes significantly affect the photosynthetic characteristics and flowering performance of two Paphiopedilum species in southwestern China (Feng et al., 2022). High-temperature stress (HS) increased canopy temperature (CT), the chlorophyll a/b ratio, leaf wax content, and anthocyanin content in pea plants, while significantly decreasing the contents of chlorophyll a, chlorophyll b, and carotenoids (Tafesse, 2018). Changes in photosynthetic pigment contents indirectly alter vegetation spectral characteristics, thereby providing a basis for monitoring and quantifying vegetation heat stress using hyperspectral techniques.

When vegetation is subjected to stress, physiological indicators change accordingly, and these changes further influence spectral responses. Spectral analysis can therefore be used to assess vegetation growth status (Kothari and Schweiger, 2022; Ren et al., 2020b). Hyperspectral techniques enable accurate estimation of vegetation growth information, and the response relationships between physiological indicators and spectral characteristics can reveal the spectral representation mechanisms underlying physiological changes in vegetation (Ren et al., 2021; Wang et al., 2021). At present, commonly used methods for spectral feature extraction include Principal Component Analysis (PCA), Maximum Noise Fraction (MNF), Linear Discriminant Analysis (LDA), Discrete Wavelet Transform (DWT), and Independent Component Analysis (ICA) (Benoit et al., 2021; Guo et al., 2021; Lan et al., 2021; Othman and Zeebaree, 2020; Zhao et al., 2022; Zhu et al., 2022). Band selection methods mainly include Competitive Adaptive Reweighted Sampling (CARS), Uninformative Variable Elimination (UVE), and the Successive Projection Algorithm (SPA) (Song et al., 2020; Yan et al., 2021; Yao et al., 2022). Frequently used modeling approaches include Support Vector Machine (SVM), Random Forest (RF), Multiple Linear Regression (MLR), and Partial Least Squares (PLS) (Cervantes et al., 2020; Shen et al., 2020; Singh et al., 2024; Sun et al., 2020). Zhang et al. (2025) conducted experiments on maize under different Cu^2+^ concentrations and applied second-order derivative (SOD) processing and continuum removal (CR) to leaf spectral reflectance. Combined with the leaf spectral detection method (LSDN), spectral information was enhanced, enabling the localization of abnormal spectral bands. The extracted spectral anomaly parameters quantitatively characterized maize leaf abnormalities under copper stress (Zhang et al., 2025). Gu et al. (2026) proposed an Extreme Gradient Boosting (XGBoost) model optimized using the Sparrow Search Algorithm (SSA) and an Extreme Learning Machine (ELM) model optimized with the Artificial Hummingbird Algorithm (AHA), both of which demonstrated superior performance in predicting wheat leaf nitrogen content (LNC) (Gu et al., 2026).

Heat stress induced by spontaneous combustion in coal gangue dumps has a significant impact on vegetation. The spontaneous combustion process affects soil pH and nutrient composition, which indirectly influences plant growth status, community composition, and ecological functions (Ran et al., 2020; Ruan et al., 2022). Numerous scholars have indicated that changes in vegetation physiological status serve as indirect indicators of combustion risk, representing an effective monitoring approach (Du and Zhang, 2009; Zhang, 2008). Researchers in Poland have identified a “natural greenhouse effect” on coal gangue dumps, characterized by the persistence of alternating vegetation during winter. Additionally, they observed plants with heights significantly exceeding historical records, potentially related to increased nitrogen availability, soil temperature, and carbon dioxide concentration (Ciesielczuk et al., 2015; Stracher et al., 2015). Field investigations revealed that alfalfa (*Medicago sativa* L.) is widely distributed across coal gangue dumps and is the dominant herbaceous species in both spontaneous combustion zones and vegetated areas. Under coal gangue dump conditions, alfalfa persists for most of the year (Zhu et al., 2021), demonstrating strong adaptability to the coal gangue environment. Under heat stress induced by spontaneous combustion, alfalfa exhibits pronounced variations in vegetation traits. In a spontaneous combustion area of a coal gangue dump in Shanxi Province, alfalfa biomass decreased by more than 300 g m^-2^ and plant height declined by over 30 cm compared with plants in unaffected areas. From the combustion zone to the safe zone, alfalfa showed significant spatial variation: the closer to the combustion zone, the larger the yellowing area at the roots, whereas alfalfa in safe zones exhibited a healthy green appearance (Ren et al., 2022a; Ren et al., 2022b). Wang et al. (2021, 2022a) conducted indoor simulation experiments to reproduce high-temperature environments associated with spontaneous combustion in coal gangue dumps and investigated heat stress effects on alfalfa. At both leaf and canopy scales, vegetation indices, correlation analysis, Lasso regression, and long short-term memory (LSTM) networks were applied. Through time-series analysis and feature selection, optimal spectral bands and vegetation indices were identified, and an SF-LSTM model was developed to discriminate heat stress intensity. The results indicated differences in sensitive bands between first-derivative spectra and original spectra, and heat stress was classified into mild, moderate, and severe levels based on stress symptoms. Furthermore, quantitative relationships between physiological indicators and spectral responses were established using alfalfa water content and fluorescence parameters (Wang et al., 2021, 2022). Ren et al. (2022a) and Ren et al. (2022b) combined unmanned aerial vehicle (UAV) imagery with field survey data and successfully estimated aboveground biomass (AGB) and plant height (PH) of alfalfa using UAV-derived red-edge chlorophyll index (CIrededge), canopy temperature depression (CTD), and canopy height model (CHM). Based on vegetation phenotypic parameters, a subsurface spontaneous combustion indication model was developed, enabling the quantification of the spatial distribution of spontaneous combustion intensity in coal gangue dumps (Ren et al., 2022b).

However, spontaneous combustion in coal gangue dumps is a complex and dynamic process, and alfalfa experiences different combustion stages throughout its life cycle. Physiological indicators and spectral responses of alfalfa vary across growth stages under spontaneous-combustion-induced heat stress. Existing studies have largely focused on vegetation physiological status at the whole life-cycle scale, with limited attention to differences in physiological states and spectral response characteristics among individual growth stages. Comparing growth-stage-dependent responses and identifying sensitive growth stages, physiological traits, and spectral features indicative of spontaneous combustion are therefore essential for improving the foundation and diagnostic capability of coal gangue dump spontaneous combustion monitoring.

To address this issue, this study selected alfalfa (*Medicago sativa* L.), a typical perennial herbaceous species used for reclamation on coal gangue dumps, as the research object. An indoor constant-temperature experiment was conducted to simulate heat stress during the early stage of spontaneous combustion in coal gangue dumps by establishing a control group and a warming treatment group. The effects of heat stress on alfalfa were analyzed based on physiological indicators, including SPAD and photosynthetic parameters, as well as canopy-level spectral characteristics. The main objectives of this study were to: (1) analyze the spectral response characteristics of alfalfa under heat stress induced by spontaneous combustion in coal gangue dumps and identify spectral bands and characteristic parameters sensitive to heat stress at different growth stages; (2) investigate the response relationships between key physiological parameters of alfalfa and spectral features to clarify the physiological mechanisms underlying spectral changes; and (3) construct and optimize spectral prediction models for monitoring heat stress in alfalfa based on sensitive spectral features, with the aim of providing technical support for the monitoring and early warning of spontaneous combustion in reclaimed coal gangue dumps.

## 2 Material and methods

### 2.1 Experimental design and data acquisition

#### 2.1.1 Experimental design

The experiment was conducted from April to July 2022 using a potted cultivation approach. Alfalfa was planted in plastic pots with a bottom diameter of 18 cm, a top diameter of 28 cm, and a height of 31.5 cm. The experimental soil consisted of a mixture of 17.5 kg of general garden soil and 0.5 kg of nutrient soil per pot. Compound fertilizer (with approximately 15%-15%-15% nitrogen-phosphorus-potassium content) was applied at a rate of 2.5 g per pot during the branching and budding stages.

**Figure 1:**
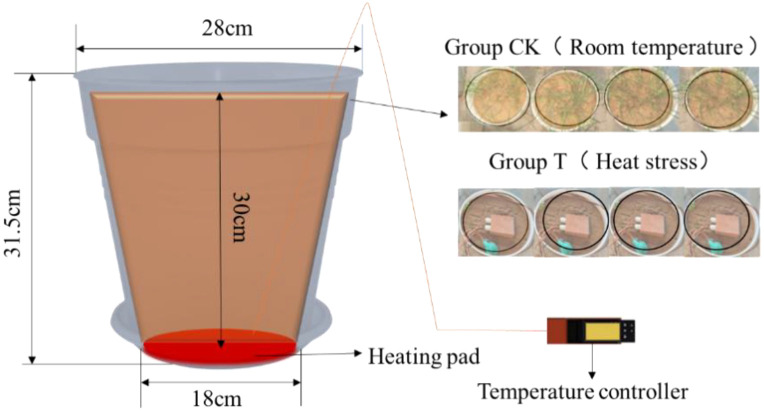
Schematic diagram of experimental design.

Alfalfa, a commonly used perennial herbaceous species for ecological restoration on coal gangue dumps, was selected for this study. The variety used was the imported ‘*Medicago sativa* L.’. The experiment included one control group (CK) and one treatment group (T), each with four replicates. The control group was allowed to grow naturally, while the treatment group was subjected to a heat source of 120 °C applied 30 cm deep in the soil to simulate the temperature stress generated during spontaneous combustion of coal gangue dumps (**Figure 1**). Soils on coal gangue dumps are highly heterogeneous and involve numerous confounding factors, such as variations in heavy metal contents and microbial communities. In addition, coal gangue dumps are typically covered with a soil layer of 30–50 cm and have already undergone ecological restoration. Conducting indoor experiments would require a large quantity of soil, and directly collecting soil samples from coal gangue dumps would cause substantial disturbance to the local ecosystem and seriously compromise restoration efforts. Therefore, homogeneous soil was used in this study.

Alfalfa was sown on April 16, 2022. Prior to sowing, uniform and full alfalfa seeds were selected, with 0.26 g sown per pot. One week after emergence of approximately half the seedlings, thinning was conducted to maintain 60 plants per pot before heat treatment. Each pot was watered with 0.5 L daily, with an additional 0.25 L applied during hot weather to ensure adequate moisture. Indoor temperature was controlled between 24 °C and 27 °C. Data were collected at intervals of four days; depending on weather conditions, measurements were advanced or delayed by one day, maintaining a final data collection interval of 3–5 days. A total of 14 measurements were conducted, all of which were completed in 2022. Specifically, measurements were carried out on May 22, May 26, June 1, June 5, and June 10, during which alfalfa was at the branching stage. Subsequent measurements were performed on June 14, June 18, June 23, June 26, and June 30, corresponding to the budding stage of alfalfa. Finally, measurements conducted on July 5, July 9, July 14, and July 18 captured the flowering stage of alfalfa.

#### 2.1.2 Data acquisition

SPAD values were measured using a SPAD-502 portable chlorophyll meter. Two pots were selected per group for measurement. Within each pot, a five-point measurement method was applied, randomly selecting the second and third fully expanded leaves from 10 uniformly growing plants as samples. As the growth stage progressed, leaves 1 and 2 from the lower part of the third fully expanded leaf were selected in later stages. Each individual leaf was measured five times, and the average value was recorded.

Photosynthetic and fluorescence parameters were measured using the LI-6800 portable photosynthesis system between 8:30 AM and 11:30 AM. After instrument calibration, environmental parameters were set for measurement (ambient CO_2_ concentration set at 400 µmol mol^-1^, temperature adjusted to 25 °C, humidity at 55%, fan speed at 10,000 rpm, and airflow rate at a conventional 500 µmol s^-1^). The leaf chamber was set to a semi-open and semi-closed state, with leaves clamped inside for measurement. Gas exchange parameters obtained included photosynthetic rate (A, µmol m^-2^ s^-1^), intercellular CO_2_ concentration (Ci, µmol mol^-1^), and stomatal conductance (gsw, mol m^-2^ s^-1^). Fluorescence parameters mainly included the effective quantum yield of PSII under light ( Fv'/Fm'), photochemical quenching coefficient (qP), and non-photochemical quenching coefficient (qN).

Canopy spectra were measured using an SVC HR-1024i full-range field spectrometer, with detailed technical specifications shown in **Table 1**. Measurements were conducted on clear and windless days between 11:00 a.m. and 2:00 p.m. Two pots per group were measured, with white reference calibration performed before each measurement. Each pot was measured using the five-point method, and each sample was measured three times; the mean value was recorded as the alfalfa canopy spectral data, covering the spectral range from 350 to 1350 nm.

**Table 1:** SVC HR-10241 technical specifications of high-resolution full-spectrum field spectrometer.

Parameter	Specification
Weight	3.8kg
Spectral Range	350nm~2500nm
Spectral Resolution	≤3.5nm@700nm ≤9.5nm@1500nm ≤6.5nm@2100nm
Spectral Sampling Width	1.5nm, 350-1000 3.8nm, 1000-1885 2.5nm, 1885-2500
Field of View	Lens:4° Optical Fiber:25°
Wavelength Accuracy	0.5nm
Wavelength Repeatability	0.1nm
Operating Environment	Temperature;-10°C~40°C Humidity:<90% Non-condensing

To reduce interference, spectral data were preprocessed using the accompanying SVC HR-1024i software. Data were first de-duplicated, then resampled, followed by smoothing of spectral curves using the Savitzky-Golay convolution smoothing method.

Data analysis and correlation analysis were conducted using Origin 2021. In addition, Origin 2021 was used to generate graphical outputs of the analytical results, and figure integration and layout were completed using Microsoft PowerPoint 2019.

### 2.2 Methods

#### 2.2.1 Extraction of sensitive spectral bands

This study employed correlation analysis to select sensitive spectral bands of alfalfa physiological indicators. Using the original spectral reflectance (R) and its first derivative reflectance (FDR) in the 350–1350 nm range as independent variables, and physiological indicators such as chlorophyll content (SPAD) and photosynthetic parameters as dependent variables. Based on Equation (1), the Pearson correlation coefficient was calculated for each wavelength band:

(1) $$r(\lambda )=\frac{\sum_{i=1}^{n}(R(\lambda_{i})-R)(Y_{i}-Y)}{\sqrt{\sum_{i=1}^{n}{\left(R(\lambda_{i})-\bar{R}\right)}^{2}\sum_{i=1}^{n}{\left(Y_{i}-\bar{Y}\right)}^{2}}}$$

Where $Y_{i}$ represents the measured physiological indicator value and $\lambda_{i}$ denotes the wavelength value.

To reduce redundancy among spectral bands and minimize the influence of measurement environment, the original and first derivative spectra in the 350–1350 nm range were used as independent variables, with physiological indicator measurements as dependent variables. Correlation analysis combined with SPA was applied to select characteristic spectral bands sensitive to physiological indicators, i.e., bands that had significant influence on physiological parameters. In this study, SPA modeling used 40 samples for calibration and 40 for prediction, resulting in the selection of different spectral feature bands for the branching stage.

SPA is a forward iterative search method that starts from one wavelength and adds a new wavelength at each iteration until the preset number of variables is reached. The goal of SPA is to select a wavelength combination with minimal redundancy in spectral information to address collinearity issues (Araújo et al., 2001; Mu et al., 2014). SPA was calculated based on Equations 2–4. The input includes the spectral data X. Initial band set K(0), the preset number of variables N, initialized iteration n=1, and spectral variables $x_{j}\in X_{j}$, j=1, …J.

1) Identification of unselected wavelength variables:

(2) S={j|1≤j≤J and j∉{k(0),.,k(n−1)}}

2) Calculate the projection of each x_j_ onto the residual vector:

(3) $$P_{X_{j}}=X_{j}-(X_{j}^{T}-X_{k(n-1)})X_{k(n-1)}{\left(X_{k(n-1)}^{T}-X_{k(n-1)}\right)}^{-1}$$

3) For all j ∈S,

(4) $$k(n)=arg(\max∥P_{X_{j}}∥,j\in S)$$

4) $x_{j}=P_{X_{j}},\;j\in S$.

5) Increment n=n+1. If n<N, return to step 1). The final variable set is denoted as s_1_={x_k(n)_|n=0,…,N-1}.

For each iteration of k(0) and N, a multiple linear regression model is established to obtain the modeling set RMSECV. The optimal values of k(0) and N correspond to the minimum RMSECV.

#### 2.2.2 Model prediction

Spectral features with strong correlation were selected from the original spectrum (OS), first derivative spectrum (FDS), triangular spectral parameters (TP), and vegetation indices (VI) as inputs for model development. Specifically, OS1 and FDS1 denote the top ten wavelength bands selected by correlation analysis from the original and first derivative spectra, while OS2 and FDS2 represent bands optimized by the SPA algorithm. For TP, indices with correlation coefficients above 0.6 were chosen (Phaneendra Kumar et al., 2024). VI was divided into two categories, with NDVI and FDNDVI serving as the optimal indices for the original and first derivative spectra, respectively. The dataset was divided into training and testing sets at a 3:1 ratio (60 training samples, 20 testing samples).

Three machine learning algorithms were applied for validation: Support Vector Regression (SVR), Random Forest Regression (RFR), and Partial Least Squares Regression (PLSR). SVR, an extension of support vector machines for nonlinear regression, balances approximation accuracy and model complexity, showing particular strength with small sample sizes and nonlinear data (Wang et al., 2023). RFR employs the Bagging technique within an ensemble of regression trees, demonstrating robustness to noise and outliers and reducing overfitting (Sun et al., 2022). PLSR combines multiple linear regression, principal component analysis, and canonical correlation analysis, excelling with limited samples by extracting richer information than classical regression methods (Wold et al., 2001).

The model calculations in this study were performed in the MATLAB R2019b environment. The kernel function of the SVR model was the radial basis function (RBF), with the penalty parameter and kernel gamma value optimized automatically by the model. The SVM function type was set to ϵ-SVR, with a loss function parameter ϵ=0.01. For the Random Forest Regression (RFR) model, the decision tree algorithm employed TreeBagger with 200 trees and a minimum leaf size of 5; the algorithm type was set to regression. Variables were randomly divided into training and testing sets at a ratio of 3:1.

Model performance was evaluated using the coefficient of determination (R^2^) and root mean square error (RMSE). A higher R^2^ and lower RMSE indicate better predictive accuracy. The calculation formulas for these metrics are as follows:

(5) $$R^{2}=1-\frac{\sum_{i=1}^{N}{\left(y_{i}-\hat{y_{i}}\right)}^{2}}{\sum_{i=1}^{N}{\left(y_{i}-\bar{y}\right)}^{2}}$$

(6) $$RMSE=\sqrt{\frac{1}{N}\sum_{i=1}^{N}{\left(y_{i}-\bar{y_{i}}\right)}^{2}}$$

Where N is the number of samples, $y_{i}$ is the actual value of the i-th sample, $\bar{y_{i}}$ is the predicted value of the i-th sample, and $\bar{y}$​ is the mean of the actual values of all samples.

## 3 Results

### 3.1 Canopy spectral and physiological variations under heat stress

#### 3.1.1 Canopy spectral changes

**Figure 2:**
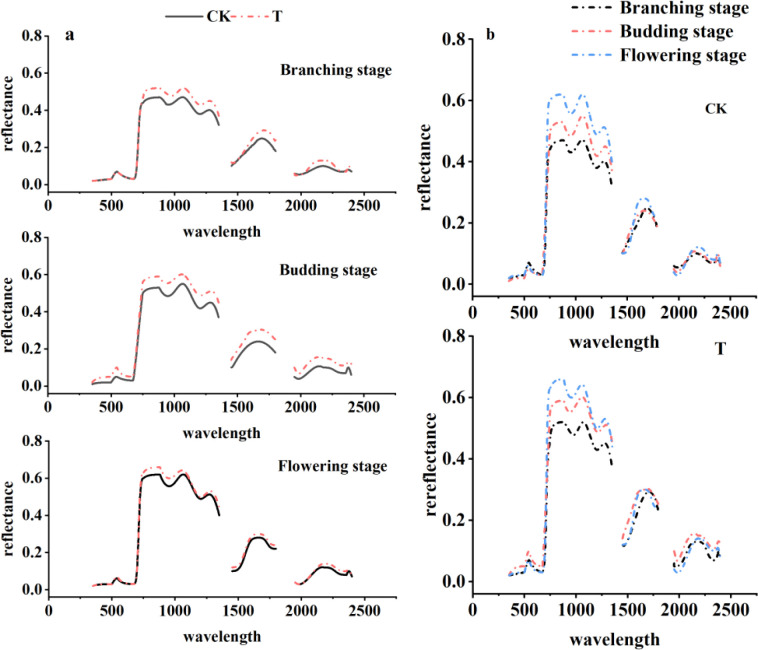
Canopy spectral variations at different growth stages (**(a)** depicted the spectral comparisons of alfalfa at different growth stages under various treatments, while **(b)** illustrated the spectral comparisons of different growth stages within the same treatment group).

The canopy spectra of alfalfa during the branching, budding, and flowering stages are shown in **Figure 2**. During the branching stage, the spectral reflectance in the visible band showed no significant difference between T and CK, but in the near-infrared (NIR) band, the reflectance of T was significantly higher than that of CK. At the budding stage, T exhibited higher reflectance than CK in the visible band, with an even more pronounced difference in the NIR band. During the flowering stage, the reflectance difference between the two groups across the full spectral range was relatively small; however, T still demonstrated higher reflectance in the NIR band. These observations indicated that heating treatment induced a marked spectral response in alfalfa growth, with the effect gradually weakening over time.

From a longitudinal comparison of spectral changes within the same treatment group across different stages, spectral reflectance of CK in the visible band exhibited a “decrease followed by an increase” trend, while the NIR plateau reflectance gradually increased. This suggests that the spectral characteristics of alfalfa vary significantly along its growth stages under natural conditions. In contrast, spectral reflectance of T in the visible band showed an “increase followed by a decrease” trend, while the NIR plateau also gradually increased. In the visible band, the green valley depth of T was notably greater than that of CK, indicating that heat stress affected chlorophyll absorption capacity. In the NIR plateau region, reflectance of T during the branching stage was significantly lower than during the budding and flowering stages, with relatively minor differences between the latter two stages. This pattern suggests that the initial increase in spectral reflectance induced by heat stress was pronounced, but as the stress duration extended, the vegetation gradually adapted and reflectance changes stabilized.

**Figure 3:**
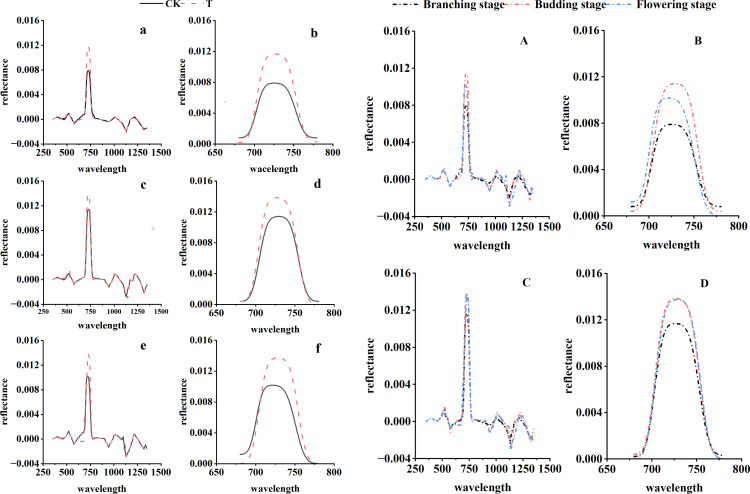
First derivative changes of canopy spectra at different growth stages (**a, c, e** represent the branching, budding, and flowering stages, respectively; **b, d, f** correspond to the respective red edge profiles; **A** denotes CK, **C** denotes T, and **B** and **D** correspond to the red edge profiles).

As shown in **Figure 3**, the first derivative of the canopy spectra exhibited the greatest fluctuations within the red edge region (680 nm–780 nm), with the maximum values in T exceeding those in CK across all three growth stages. The red edge area in T was significantly larger than that in CK during all stages. During the branching stage, the red edge position for CK was at 725 nm, while T showed a red shift to 726 nm. At the budding stage, red edge position of CK shifted to 729 nm, whereas T exhibited a blue shift to 727 nm. During the flowering stage, red edge of CK was at 721 nm, and T showed a pronounced red shift to 728 nm. Within the same treatment group, red edge position of CK changed from an initial red shift to a subsequent blue shift, which is consistent with normal plant growth patterns. The red edge amplitude followed the order: budding stage > flowering stage > branching stage, while the red edge area ranked flowering stage > budding stage > branching stage. Red edge position of T also shifted from red to blue, with the red edge amplitude and area ranking as budding stage > flowering stage > branching stage. The effects of heat stress on the first derivative spectral changes in alfalfa showed no significant difference between the budding and flowering stages.

#### 3.1.2 Physiological parameter changes

The amplitude of variation was calculated using Equation 7:

(7) $$\eta =\frac{\left[V(T)-V(CK)\right]}{V(CK)}*100\%$$

**Figure 4:**
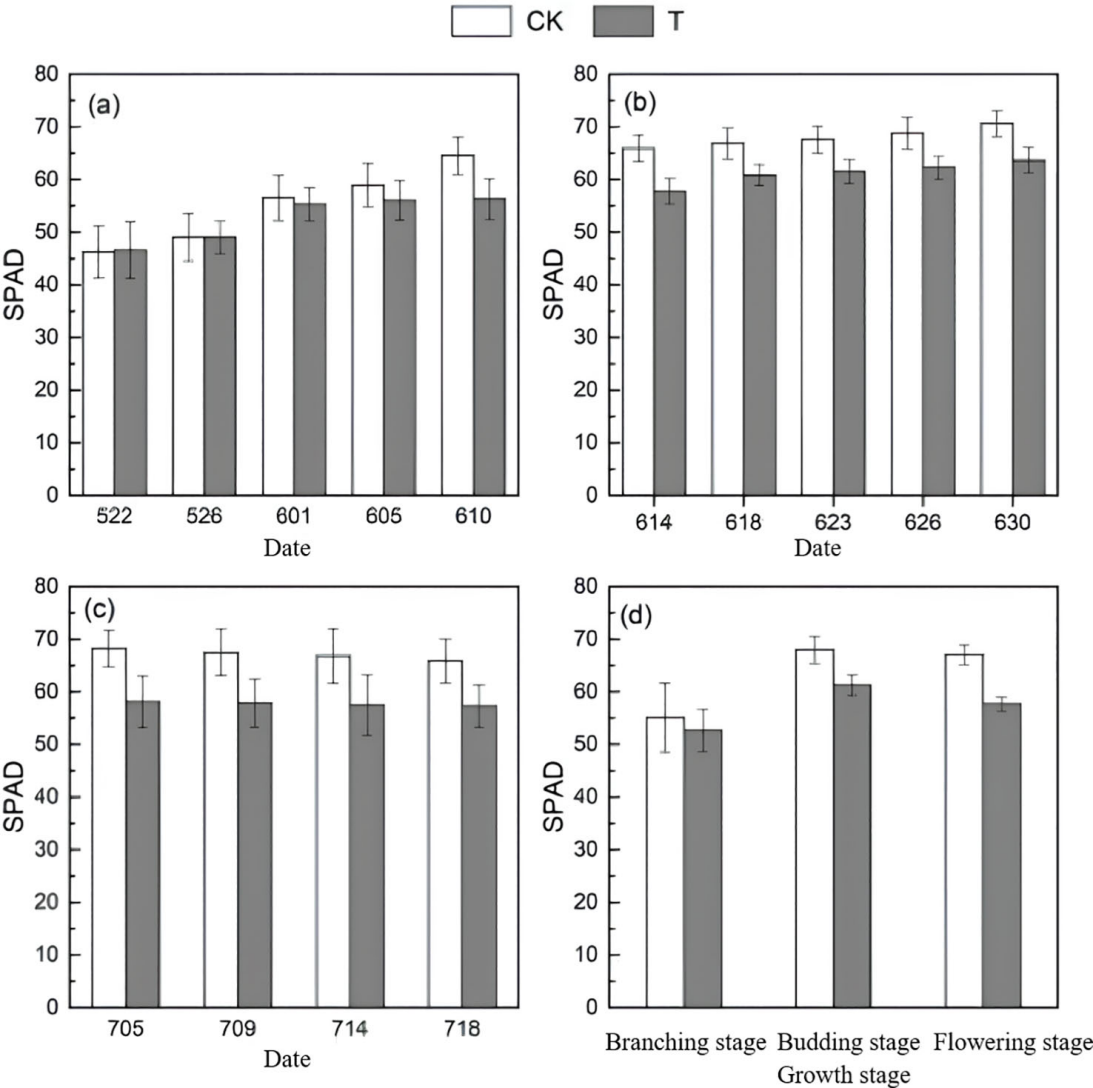
SPAD variation trends at different growth stages (**(a)** branching stage **(b)** budding stage **(c)** flowering stage **(d)** changes across all three stages).

As shown in **Figures 4a–c**, except for an amplitude increase of 0.65% on May 22, the variation amplitude of chlorophyll content (SPAD) in T was consistently lower than that in CK during the branching, budding, and flowering stages, with all exhibiting an increasing trend. In **Figure 4d**, the overall trend across the three growth stages shows an initial increase followed by a decrease, indicating that the plant growth pattern remained unchanged. Comparing SPAD between CK and T, the amplitude of variation in T was lower at each stage, suggesting that heat stress exerted an inhibitory effect on the relative chlorophyll content. It can be inferred that temperature partially limits alfalfa growth. Furthermore, in **Figures 4a–c**, SPAD increments exhibited a pattern of first rising and then falling, with increase of CK generally exceeding that of T, indicating that heat stress restricted the rate of SPAD increase to some extent.

**Figure 5:**
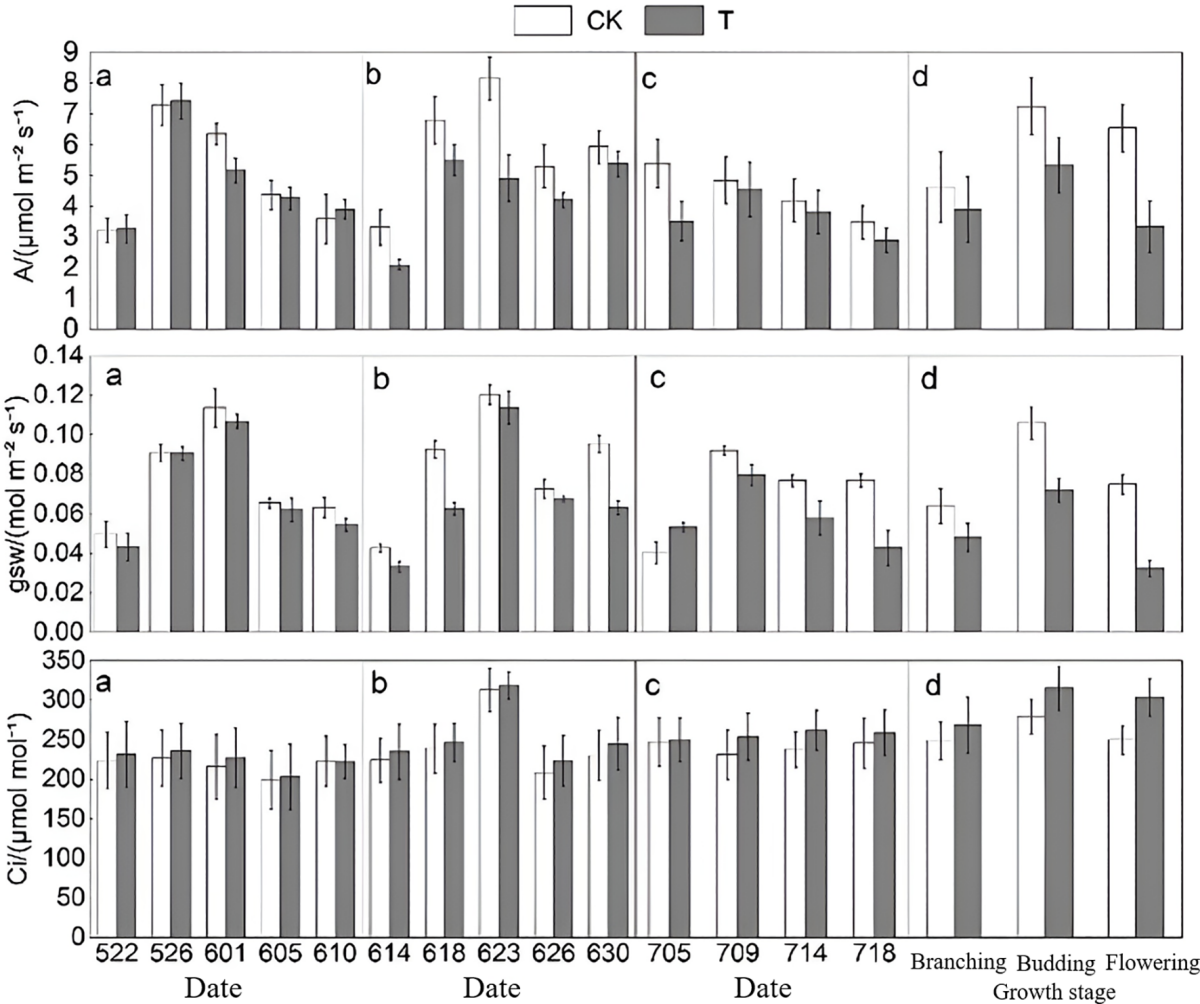
Trends in gas exchange parameters at different growth stages (**a–c** represent the branching, budding, and flowering stages, respectively; **d** shows the overall mean data across the three stages. A denotes photosynthetic rate (A/µmol m^-2^ s^-1^), gsw represents stomatal conductance (gsw/mol m^-2^ s^-1^), and Ci indicates intercellular CO_2_ concentration (Ci/µmol mol^-1^)).

As shown in **Figure 5**, the photosynthetic rate (A) variation trend in T was generally consistent with that of CK. Except during the branching stage, where the amplitude of change in T exceeded that of CK, the variation amplitudes on other dates were all lower than those in CK. During the branching stage, photosynthetic rate of T initially increased and then decreased; in the budding stage, it first increased, then decreased, and increased again; during the flowering stage, it rose initially and then declined. Overall, the photosynthetic rate decreased by 3.32%, 25.17%, and 17.46% during the branching, budding, and flowering stages, respectively, indicating that heat stress to some extent reduces photosynthetic intensity and slows the photosynthetic rate.

Stomatal conductance (gsw) measurements were consistently lower in T compared to CK across all stages. From branching to flowering, the decrease in stomatal conductance became more pronounced, though the overall trend in both groups was similar—initially increasing and then decreasing. In this study, higher stomatal conductance allows more CO_2_ to enter cells, resulting in lower intercellular CO_2_ concentration (Ci) and higher net photosynthetic rate. When stomata close, Ci also decreases. In contrast to photosynthetic rate and stomatal conductance, T exhibited consistently lower Ci values compared to CK at all measured stages. Across branching, budding, and flowering stages, Ci showed an overall increasing trend, with the average increase growing over time, consistent with the duration of heat stress.

**Figure 6:**
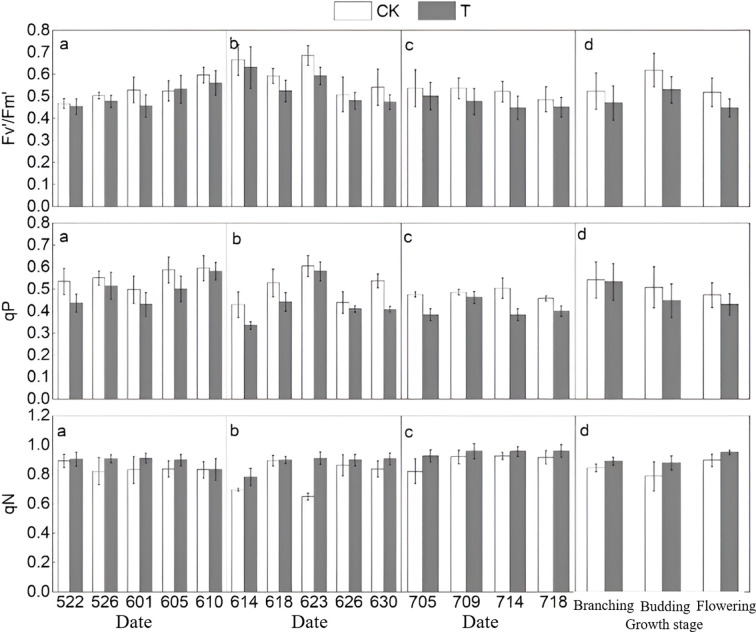
Trends in fluorescence parameters at different growth stages (**a–c** represent the branching, budding, and flowering stages, respectively; **d** shows the overall mean data across the three stages. Fv'/Fm' denotes the PSII photochemical efficiency under light, qP represents the photochemical quenching coefficient, and qN indicates the non-photochemical quenching coefficient).

The comparison results of Fv'/Fm' and qP show that the values in T are consistently lower than those in CK (**Figure 6**). The overall trend of Fv'/Fm' first rises and then declines, with the mean changes across the three stages consistent with multiple measurements, showing decreases of 5.39%, 9.73%, and 9.92% at the branching, budding, and flowering stages, respectively. For qP, T values are also lower than those of CK, with an overall trend from branching to flowering stages consistent with CK, and the mean decrease progressively increasing at 10.83%, 11.37%, and 15.20% respectively. In contrast to Fv'/Fm' and qP, the qN values in T were consistently higher than those in CK, increasing by 5.57%, 11.56%, and 5.99% at branching, budding, and flowering stages respectively, showing a trend of first increasing and then decreasing.

Based on the trends of photosynthetic and fluorescence parameters throughout the entire growth period, the most significant changes among the six photosynthetic parameters are observed in gsw, qP, A, and qN. Changes in gas exchange parameters are more pronounced, with qP and qN showing considerable variation among fluorescence parameters. From these results, sensitive photosynthetic parameters at the branching and flowering stages include gas exchange parameter gsw and fluorescence parameter qP, whereas at the budding stage, sensitive parameters include gas exchange parameter A and fluorescence parameter qN.

### 3.2 Sensitive spectral bands and features of physiological indicators across growth stages

#### 3.2.1 Branching stages

**Figure 7:**
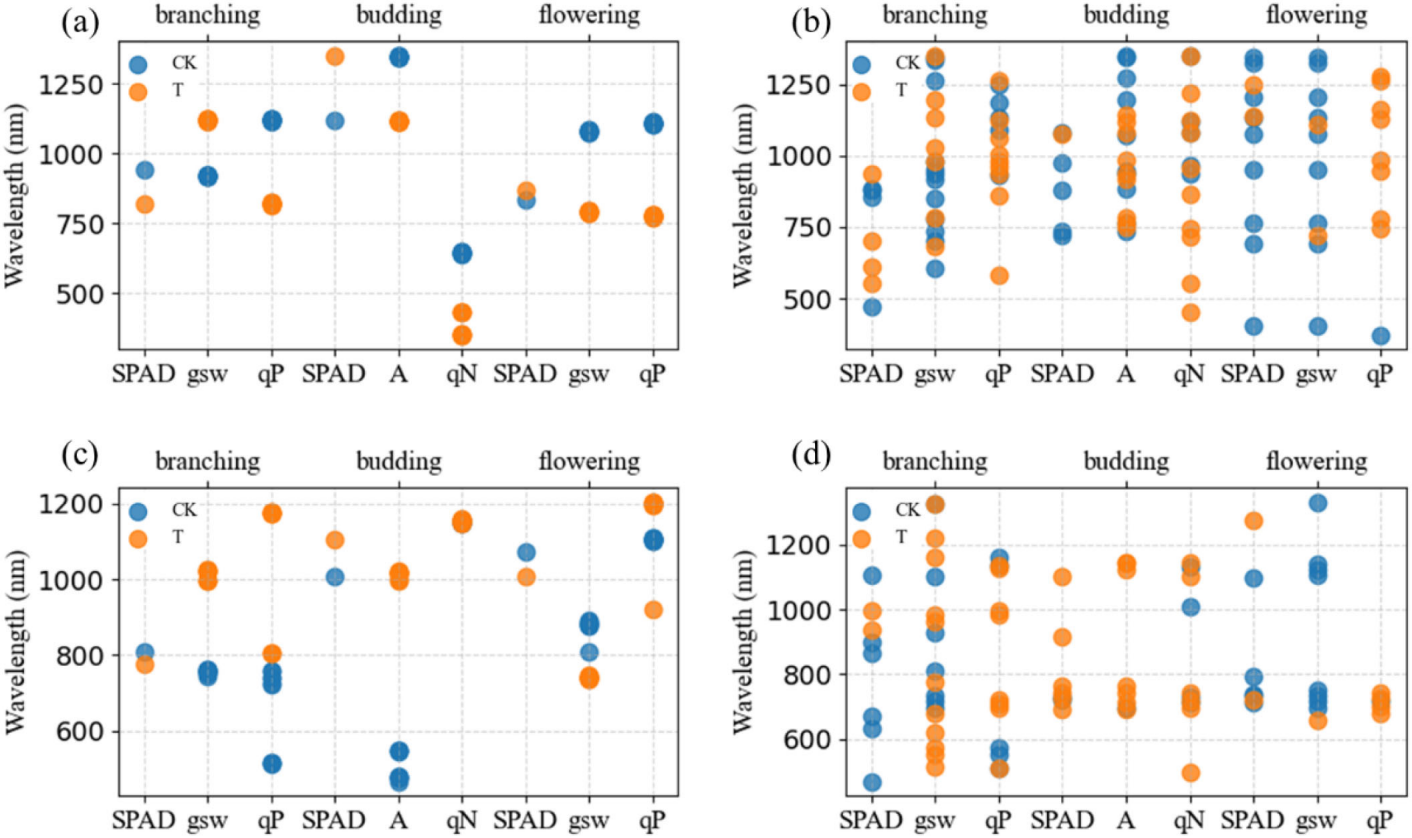
Sensitive bands at different growth stages (**a** original spectra – correlation analysis, **b** original spectra – SPA, **c** first-derivative spectra – correlation analysis, **d** first-derivative spectra – SPA).

The spectral characteristic bands at the branching stage were shown in **Figure 7**. In the original spectra, the qP characteristic bands in CK were similar to those of gsw, both being concentrated in the near-infrared reflectance plateau. In T, the gsw bands shifted toward longer wavelengths compared with CK, while the qP bands shifted toward shorter wavelengths. In the first-derivative spectra, the sensitive bands of both gsw and qP in T shifted toward longer wavelengths relative to CK.

A total of 20 spectral red-edge parameters were selected for analysis, comprising 10 positional parameters, 3 area parameters, and 7 red-edge index parameters. Previous studies have demonstrated that these parameters are highly sensitive to variations in plant growth and physiological status (Guo et al., 2018; Xie et al., 2018).

**Figure 8:**
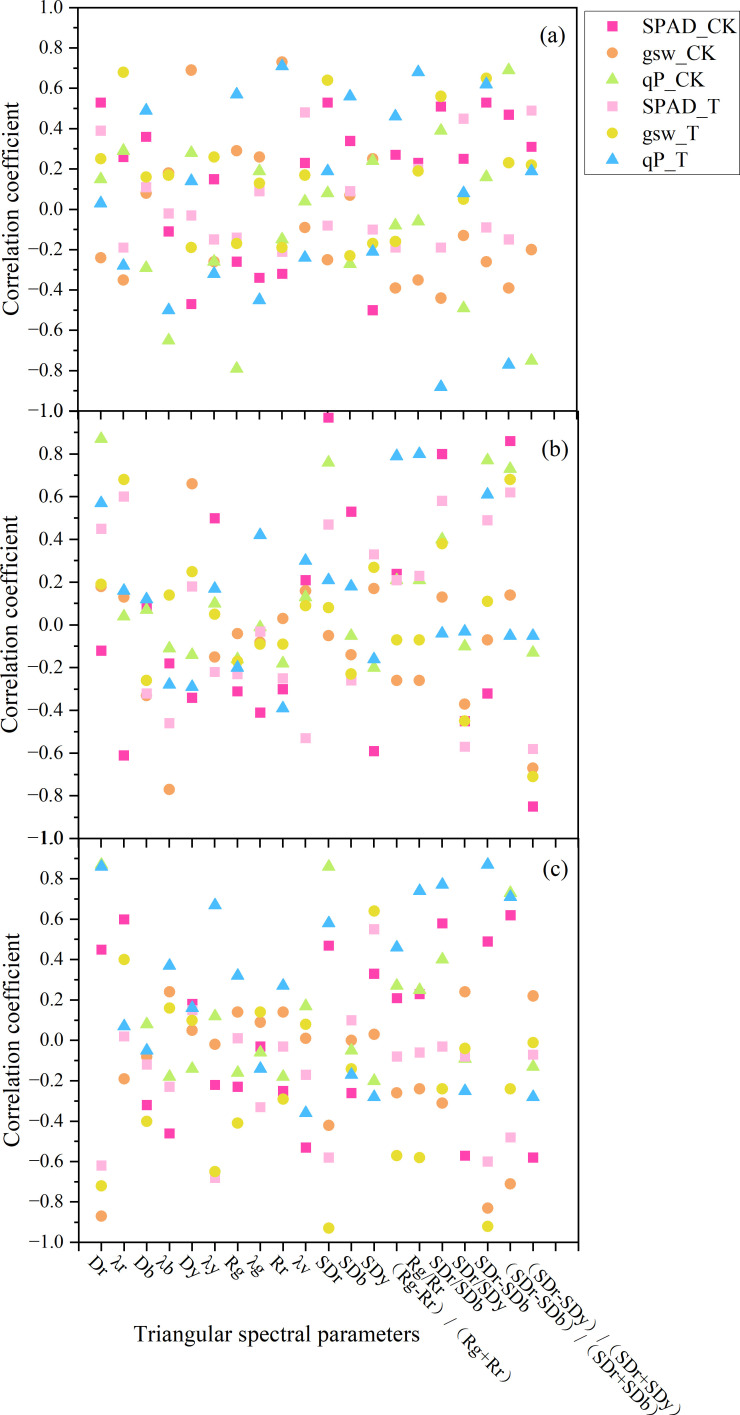
Correlation coefficients between three-edge parameters and physiological indices (**a** branching stage; **b** budding stage; **c** flowering stage). Notes (same below): *D_r_* — red-edge amplitude; *λ_r_* — red-edge position; *D_b_* — blue-edge amplitude; *λ_b_* — blue-edge position; *D_y_* — yellow-edge amplitude; *λ_y_* — yellow-edge position; *R_g_* — green peak reflectance; *λ_g_* — green peak position; *R_r_* — red valley amplitude; *λ_v_* — red valley position; *SD_r_* — red-edge area; *SD_b_* — blue-edge area; *SD_y_* — yellow-edge area; (*R_g_* – *R_r_*)/(*R_g_* + *R_r_*) — normalized difference of green peak and red valley reflectance; *R_g_*/*R_r_* — ratio of green peak to red valley reflectance; *SD_r_*/*SD_b_* — ratio of red-edge area to blue-edge area; *SD_r_*/*SD_y_* — ratio of red-edge area to yellow-edge area; *SD_r_* – *SD_b_* — difference between red-edge area and blue-edge area; (*SD_r_* – *SD_b_*)/(*SD_r_* + *SD_b_*) — normalized difference of red-edge area and blue-edge area; (*SD_r_* – *SD_y_*)/(*SD_r_* + *SD_y_*) — normalized difference of red-edge area and yellow-edge area.

During the branching stage (**Figure 8a**), the chlorophyll content (SPAD) in CK exhibited the strongest correlation with SD_r_-SD_b_ (r = 0.53, p < 0.05), followed by D_r_、SD_r_、SD_r_/SD_b_、SD_y_、(SD_r_-SD_b_)/(SD_r_+SD_b_) and D_y_, with correlation coefficients of 0.53, 0.53, 0.51, −0.50, 0.47, and −0.47, respectively (p < 0.05 for all). In T, the parameter most strongly associated with SPAD was (SD_r_-SD_y_)/(SD_r_+SD_y_) (r = 0.49, p < 0.05), followed by λ_v_ (r = 0.48) and SD_r_/SD_y_ (r = 0.45). Overall, correlations in CK were stronger than those in T.

For photosynthetic parameters during the branching stage, gsw in CK showed extremely significant correlations with D_y_ (r = 0.69) and R_f_ (r = 0.73). qP was also extremely significantly correlated with λ_b_ (r = −0.65), R_g_ (r = −0.79), (SD_r_-SD_b_)/(SD_r_+SD_b_) (r = −0.69), and (SD_r_-SD_y_)/(SD_r_+SD_y_) (r = −0.75). In T, gsw responded most strongly to λ_r_ (r = 0.68), SD_r_ (r = 0.64), SD_r_/SD_b_ (r = 0.56), and SD_r_-SD_b_ (r = 0.65). qP exhibited the highest correlations with R_g_ (r = 0.57), R_r_ (r = 0.71), R_g_/R_r_ (r = 0.68), SD_r_/SD_b_ (r = −0.88), SD_r_-SD_b_ (r = 0.62), and (SD_r_-SD_b_)/(SD_r_+SD_b_) (r = −0.77).

Overall, the three indicators (SPAD, gsw, and qP) generally exhibited stronger correlations with red-edge parameters in CK than in T. Among these, photosynthetic parameters displayed stronger associations than SPAD. Within T, qP demonstrated the highest sensitivity to temperature changes, suggesting its potential as a key indicator for assessing heat stress responses.

During the branching stage, the SPAD-sensitive bands were primarily distributed in the infrared region (**Table 2**). In CK, the SPAD values from the original spectra showed the strongest correlation with DVI (553, 705) (r = 0.80), while the SPAD values from the first-derivative spectra were most strongly correlated with FDNDVI (1083, 922) (r = 0.87). In T, the SPAD values from the original spectra exhibited the strongest correlations with NDVI (891, 890) and DVI (891, 890) (both r = 0.50), whereas the SPAD values from the first-derivative spectra were most strongly correlated with FDDVI (849, 844) (r = 0.81).

**Table 2:** Branching stage: optimal vegetation index bands and corresponding correlation coefficients.

Physiological indicators		SPAD		gsw		qP	
Group		CK	T	CK	T	CK	T
RVI	Wavelength combination	779,777	891,890	691,630	1044,968	827,828	1002,981
	Correlation coefficient	0.72	0.49	0.65	0.53	0.49	0.83
NDVI	Wavelength combination	779,777	819,890	691,630	1044,968	827,828	1002,981
	Correlation coefficient	0.73	0.5	0.65	0.53	0.49	0.83
DVI	Wavelength combination	553,705	891,890	772,780	1088,958	827,829	997,981
	Correlation coefficient	0.8	0.5	0.59	0.53	0.51	0.81
FDRVI	Wavelength combination	800,987	457,1109	991,1275	844,848	939,1273	1264,805
	Correlation coefficient	0.84	0.7	0.76	0.73	0.77	0.84
FDNDVI	Wavelength combination	1083,922	1110,1006	937,628	844,849	1247,847	982,1002
	Correlation coefficient	0.87	0.78	0.79	0.73	0.76	0.85
FDDVI	Wavelength combination	850,823	849,844	956,966	988,372	891,890	982,918
	Correlation coefficient	0.85	0.81	0.68	0.71	0.71	0.9

For photosynthetic parameters in the branching stage, the optimal index for gsw in CK was the first derivative spectral index FDNDVI (937, 628) (r = 0.79), while the optimal index for qP was FDRVI (939, 1273) (r = 0.77). In T, the optimal indices for gsw were FDNDVI (844, 849) and FDRVI (844, 848), both with r = 0.73, and the optimal index for qP was FDDVI (982, 918) (r = 0.90). The two photosynthetic parameters exhibited contrasting spectral responses between treatments: gsw showed stronger spectral responsiveness in CK compared to T, whereas qP was more responsive in T than in CK.

#### 3.2.2 Budding stage

In the budding stage, the sensitive wavelengths of A in the original spectrum for T shifted toward shorter wavelengths compared to CK, with qN showing the same shift direction as A; however, in the first derivative spectrum, the sensitive wavelengths of both A and qN for T shifted toward longer wavelengths relative to CK (**Figure 7**).

During the budding stage (**Figure 8b**), the spectral feature parameter that was most strongly correlated with chlorophyll content in CK was SDr, with a correlation coefficient of 0.97 (p < 0.01). Other parameters that showed high correlations included (SDr-SDb)/(SDr+SDb), (SDr-SDy)/(SDr+SDy), SDr/SDb, λr, and SDy, with respective correlation coefficients of 0.86, 0.85, 0.80, 0.61, and 0.59, all at a highly significant level. For T during the budding stage, the spectral parameter most correlated with chlorophyll content was SDr-SDb, with a correlation coefficient of 0.63 (p < 0.01). Other parameters that had notable correlations were Dr and λb, with coefficients of 0.56 and -0.54, significant at the 0.05 level.

For CK in the budding stage, A showed highly significant correlations with λ_b_, D_y_, and (SD_r_-SD_y_)/(SD_r_+SD_y_), with coefficients of -0.77, 0.66, and -0.67, respectively. qN was highly correlated with D_r_, SD_r_, SD_r_-SD_b_, and (SD_r_-SD_b_)/(SD_r_+SD_b_), with coefficients of 0.87, 0.76, 0.77, and 0.73. In T, A was highly correlated with λ_r_, (SD_r_-SD_b_)/(SD_r_+SD_b_), and (SD_r_-SD_y_)/(SD_r_+SD_y_), with coefficients of 0.68, 0.68, and -0.71. qN showed strong correlations with D_r_, (R_g_-R_r_)/(R_g_+R_r_), R_g_/R_r_, and SD_r_-SD_b_, with coefficients of 0.57, 0.79, 0.80, and 0.61.

During the budding stage, the SPAD-sensitive bands in CK were primarily concentrated in the infrared region (**Table 3**), whereas those in T were more widely dispersed. In CK, the SPAD values derived from the original spectra exhibited strong correlations with RVI (1150,1347) and NDVI (1151,1347), with correlation coefficients of 0.77. The first derivative spectra showed the highest correlation with FDRVI (1161,1156), with a coefficient of 0.82. In T, SPAD values from the original spectra were most strongly correlated with RVI (683,666) and NDVI (683,666) (correlation coefficient = 0.55), while the first derivative spectra were most strongly correlated with FDNDVI (1170,388) (correlation coefficient = 0.82).

**Table 3:** Budding stage: optimal vegetation index bands and correlation coefficients.

Physiological indicators		SPAD		A		qN	
Group		CK	T	CK	T	CK	T
RVI	Wavelength combination	1150,1347	683,666	990,972	1261,1147	971,987	659,660
	Correlation coefficient	0.77	0.55	0.85	0.52	0.86	0.57
NDVI	Wavelength combination	1150,1347	683,666	990,972	1261,1147	971,987	659,660
	Correlation coefficient	0.77	0.55	0.85	0.52	0.86	0.57
DVI	Wavelength combination	1158,1172	909,908	991,972	1245,1152	972,987	668,678
	Correlation coefficient	0.71	0.45	0.85	0.53	0.86	0.48
FDRVI	Wavelength combination	1161,1156	479,980	980,455	778,1266	927,898	671,892
	Correlation coefficient	0.82	0.75	0.8	0.71	0.93	0.73
FDNDVI	Wavelength combination	918,760	1170,388	984,411	947,800	1315,1300	599,822
	Correlation coefficient	0.8	0.82	0.82	0.77	0.91	0.76
FDDVI	Wavelength combination	1166,372	462,46	984,1090	889,893	898,660	973,916
	Correlation coefficient	0.79	0.76	0.85	0.61	0.89	0.72

For the photosynthetic parameter A in CK, all four vegetation indices except FDNDVI and FDRVI showed high correlations (r = 0.85) with optimal bands of NDVI (990,972), DVI (991,972), RVI (990,972), and FDDVI (984,1090). In T, the optimal index was FDNDVI (947,800), with a correlation coefficient of 0.77. For the photosynthetic parameter qN, CK exhibited the highest correlation with FDRVI (927,898) (r = 0.93), while in T the highest correlation was with FDNDVI (599,822) (r = 0.76). Overall, the spectral responses of A and qN during the budding stage were stronger in CK than in T, and vegetation indices derived from first derivative spectra showed better performance than those from the original spectra.

#### 3.2.3 Flowering stage

Based on correlation analysis and the SPA algorithm, the sensitive bands of physiological indices during the flowering stage were identified (**Figure 7**). For the raw spectra, the sensitive bands of gsw in T shifted toward shorter wavelengths compared with CK, and qP exhibited a similar shift direction to gsw. In the first derivative spectra, the sensitive bands of gsw in T also shifted toward shorter wavelengths relative to CK, whereas qP shifted in the opposite direction, toward longer wavelengths.

During the flowering stage (**Figure 8c**), the spectral characteristic parameter most strongly correlated with chlorophyll content in CK was (SD_r_−SD_b_)/(SD_r_ + SD_b_), with a correlation coefficient of 0.62 (p < 0.01). Other parameters significantly correlated at the 0.01 level included λ_r_, SD_r_/SD_b_, (SD_r_-SD_y_)/(SD_r_+SD_y_), and SD_r_/SD_y_, with coefficients of 0.60, 0.58, 0.57, and 0.57, respectively. Parameters correlated at the 0.05 level included λ_y_, SD_r_-SD_b_,SD_r_, λ_b_, and D_r_, with coefficients of 0.53, 0.50, 0.47, 0.46, and 0.46, respectively. In the branching stage, the spectral parameter most strongly correlated with chlorophyll content in T was λ_y_, with a correlation coefficient of −0.68 (p < 0.01).

During the flowering stage, in CK, gsw showed extremely significant correlations with D_r_, SD_r_-SD_b_, and (SD_r_-SD_b_)/(SD_r_+SD_b_), with correlation coefficients of −0.87, −0.83, and −0.81, respectively. qP was extremely significantly correlated with D_r_, SD_r_, SD_r_ − SD_b_, and (SD_r_ − SD_b_)/(SD_r_ + SD_b_), with coefficients of 0.87, 0.86, 0.87, and 0.73, respectively. In T, gsw was extremely significantly correlated with D_r_, λ_y_, SD_r_, SD_y_, (R_g_ − R_r_)/(R_g_ + R_r_), R_g_/R_r_, and SD_r_-SD_b_, with correlation coefficients of −0.72, −0.65, −0.93, 0.64, −0.57, −0.58, and −0.92, respectively. qP exhibited extremely significant correlations with D_r_, λ_y_, SD_r_, R_g_/R_r_, SD_r_/SD_b_, SD_r_-SD_b_, and (SD_r_-SD_b_)/(SD_r_+SD_b_), with coefficients of 0.86, 0.67, 0.58, 0.74, 0.77, 0.87, and 0.71, respectively.

During the flowering stage, the SPAD-sensitive bands were mainly distributed in the infrared region (**Table 4**). In CK, the SPAD values from the original spectra showed a strong correlation with DVI (920, 919) (r = 0.69), while the SPAD values from the first derivative spectra exhibited the same correlation coefficient (r = 0.80) with FDDVI (862, 861), FDNDVI (862, 861), and FDRVI (913, 919). In T, the SPAD values from the original spectra were most strongly correlated with DVI (977, 975) (r = 0.66), whereas the first derivative spectra showed the highest correlation with FDDVI (862, 861) (r = 0.73).

**Table 4:** Flowering stage: optimal vegetation index bands and correlation coefficients.

Physiological indicators		SPAD		gsw		qP	
Group		CK	T	CK	T	CK	T
RVI	Wavelength combination	920,919	977,975	1273,1272	695,350	1337,726	1328,723
	Correlation coefficient	0.69	0.66	0.74	0.82	0.53	0.62
NDVI	Wavelength combination	920,919	963,962	1273,1272	697,350	1337,726	1327,723
	Correlation coefficient	0.68	0.6	0.74	0.83	0.53	0.62
DVI	Wavelength combination	920,919	963,962	916,915	585,584	1320,718	870,868
	Correlation coefficient	0.68	0.6	0.72	0.78	0.72	0.59
FDRVI	Wavelength combination	862,861	862,861	922,875	1270,901	594,959	902,891
	Correlation coefficient	0.8	0.73	0.87	0.84	0.71	0.74
FDNDVI	Wavelength combination	862,861	862,861	416,934	1001,1004	595,594	505,561
	Correlation coefficient	0.8	0.69	0.9	0.84	0.78	0.72
FDDVI	Wavelength combination	913,919	913,919	646,640	588,595	657,492	918,932
	Correlation coefficient	0.8	0.69	0.84	0.85	0.78	0.71

For the photosynthetic parameter gsw, the spectral response of T was generally superior to that of CK. The highest correlation coefficient for both groups (r = 0.90) was observed in CK for the first derivative spectral index FDNDVI (416, 934), while T achieved its highest correlation (r = 0.85) with FDDVI (588, 595). For the photosynthetic parameter qP, the optimal spectral indices in CK were FDNDVI (595, 594) and FDDVI (657, 492), both with correlation coefficients of 0.78, whereas in T the optimal spectral index was FDRVI (902, 891) with a correlation coefficient of 0.74. Overall, the gsw spectral response in CK was superior to that of qP.

### 3.3 Model prediction of physiological indicators at different growth stages

**Figure 5** shows that during the branching stage, the overall prediction performance of the three models ranked as SVR > PLSR > RFR, with R^2^ values for both training and testing sets ranging between 0.6 and 0.9, and training set values generally higher than those of the testing set. The most frequently selected optimal feature was the first-derivative spectral vegetation index FDNDVI, followed by TP and NDVI. Among the three physiological indicators (SPAD, gsw, and qP), the SVR model achieved the best prediction for gsw; the RFR model performed best for qP; and the PLSR model yielded the highest accuracy for SPAD. Overall, the prediction results for alfalfa under normal growth and heat stress conditions showed no significant differences, indicating that early-stage heat stress has limited impact and physiological traits during the branching stage are insufficient to serve as distinguishing markers.

For the budding stage, the prediction performance of the untreated group was better than that of the treated group across the three models. In the SVR model, R² values exceeded 0.9 in some cases, with prediction accuracy ranking as SVR > PLSR > RFR. The most frequently selected optimal feature was FDNDVI, followed by NDVI and FDS2. Within the SVR model, the best predicted physiological indicator was A, with NDVI as the optimal feature; for the RFR model, SPAD showed the best prediction, with TP as the optimal feature; and for the PLSR model, qN was best predicted, with FDNDVI as the optimal feature. No clear pattern was observed between CK and T in prediction performance, and RMSE values were generally high.

The model prediction results for the flowering stage showed R² values ranging from 0.6 to 1. Overall, the prediction performance ranked as SVR > RFR > PLSR. The most frequently occurring optimal feature remained FDNDVI, followed by NDVI. Within the SVR model, the best predicted physiological indicator was qP, with MDVI as the optimal feature. For the RFR model, gsw had the best prediction performance, with FDNDVI as the optimal feature. In the PLSR model, gsw also showed the best prediction, with NDVI as the optimal feature.

Based on a comprehensive analysis of **Tables 5**–**7**, from the perspective of growth stages, prediction performance evaluated by R² indicates that the budding stage outperforms both the flowering and branching stages. The branching stage represents the initial phase of heat stress, during which the impact on alfalfa growth is not significant. However, as heat stress duration increases, its effects become more pronounced. Regarding prediction models, across different growth stages and physiological indicators, all three models show that SVR consistently outperforms RFR and PLSR. The RFR model provides the best prediction for SPAD, while the SVR model yields the best predictions for photosynthetic parameters. Among the best prediction results from branching to flowering stages, the most frequently appearing index is FDNDVI, followed by NDVI, indicating that spectral vegetation indices are the optimal predictive variables.

**Table 5:** Branching stage: optimal spectral features of physiological indicator.

Model	Physiological indicator	Treatment	CK				Treatment	T			
			Training set		Testing set			Training set		Testing set	
		Feature	R_c1_^2^	RMSE_c1_	R_c2_^2^	RMSE_c2_	Feature	R_t1_^2^	RMSE_t1_	R_t2_^2^	RMSE_t2_
SVR	SPAD	FDNDVI	0.83	3.06	0.79	4.08	FDNDVI	0.76	3.55	0.74	2.33
	gsw	NDVI	0.84	0.02	0.76	0.03	NDVI	0.88	0.01	0.76	0.03
	qP	FDS2	0.84	0.04	0.73	0.05	FDNDVI	0.84	0.04	0.79	0.05
RFR	SPAD	FDS2	0.74	2.73	0.68	4.25	FDNDVI	0.69	3.27	0.66	3.73
	gsw	FDS2	0.68	0.03	0.74	0.03	TP	0.74	0.02	0.69	0.02
	qP	FDNDVI	0.78	0.04	0.69	0.05	TP	0.81	0.05	0.74	0.05
PLSR	SPAD	TP	0.70	2.47	0.60	2.98	FDNDVI	0.85	3.14	0.75	3.93
	gsw	FDNDVI	0.75	0.04	0.66	0.05	FDNDVI	0.73	0.04	0.63	0.05
	qP	OS2	0.71	0.05	0.64	0.06	TP	0.72	0.05	0.65	0.06

**Table 6:** Budding stage: optimal spectral features of physiological indicator.

Model	Physiological indicator	Treatment	CK				Treatment	T			
			Training set		Testing set			Training set		Testing set	
		Feature	R_c1_^2^	RMSEc_1_	R_c2_^2^	RMSE_c2_	Feature	R_t1_^2^	RMSE_t1_	R_t2_^2^	RMSE_t2_
SVR	SPAD	FDNDVI	0.90	1.45	0.77	1.93	FDNDVI	0.86	3.04	0.77	3.50
	A	NDVI	0.91	1.56	0.85	1.67	NDVI	0.96	0.60	0.88	1.39
	qN	FDNDVI	0.94	0.03	0.81	0.06	FDNDVI	0.95	0.03	0.68	0.07
RFR	SPAD	FDS1	0.76	2.90	0.68	4.25	TP	0.83	2.25	0.70	2.38
	A	FDNDVI	0.80	1.39	0.76	2.23	FDS2	0.71	2.46	0.63	2.03
	qN	FDS2	0.81	0.09	0.70	0.1	NDVI	0.79	0.07	0.65	0.08
PLSR	SPAD	FDS1	0.70	2.84	0.93	1.32	FDNDVI	0.85	2.76	0.73	3.92
	A	FDNDVI	0.83	1.01	0.66	2.00	FDNDVI	0.76	1.47	0.65	2.13
	qN	FDNDVI	0.87	0.01	0.76	0.06	FDNDVI	0.89	0.01	0.72	0.07

**Table 7:** Flowering stage: optimal spectral features of physiological indicator.

Model	Physiological indicator	Treatment	CK				Treatment	T			
			Training set		Testing set			Training set		Testing set	
		Feature	R_c1_^2^	RMSEc_1_	R_c2_^2^	RMSE_c2_	Feature	R_t1_^2^	RMSE_t1_	R_t2_^2^	RMSE_t2_
SVR	SPAD	TP	0.76	2.98	0.76	2.55	NDVI	0.71	2.94	0.68	2.83
	gsw	FDNDVI	0.89	0.01	0.86	0.02	FDNDVI	0.88	0.01	0.79	0.02
	qP	TP	0.88	0.04	0.75	0.06	NDVI	0.96	0.02	0.87	0.04
RFR	SPAD	NDVI	0.70	3.21	0.69	2.92	NDVI	0.69	3.06	0.68	3.05
	gsw	FDNDVI	0.79	0.01	0.73	0.02	FDNDVI	0.83	0.01	0.71	0.01
	qP	FDNDVI	0.68	0.06	0.62	0.07	NDVI	0.77	0.06	0.68	0.06
PLSR	SPAD	FDNDVI	0.74	2.76	0.64	1.49	FDNDVI	0.68	3.31	0.63	3.33
	gsw	OS2	0.77	0.02	0.66	0.02	NDVI	0.84	0.01	0.72	0.01
	qP	FDNDVI	0.79	0.05	0.66	0.06	FDNDVI	0.66	0.02	0.59	0.06

## 4 Discussion

### 4.1 Effects of heat stress on alfalfa growth

Vegetation physiological traits and spectral characteristics undergo changes under abiotic stress (Wahid et al., 2007). Numerous studies have demonstrated that variations in these traits and spectral features caused by stress can serve as important indicators to assess the extent of abiotic stress on vegetation, as well as to evaluate the stress levels experienced by plants. This study focused on alfalfa, a typical herbaceous species used for reclamation on coal gangue dumps, conducting controlled indoor heat stress experiments. Physiological parameters and hyperspectral characteristics of alfalfa at different growth stages under natural conditions and high-temperature treatments were measured to investigate the response of alfalfa to heat stress. Sensitive spectral features were selected to establish physiological parameter models for validation.

Photosynthesis is the fundamental source of energy and material basis for plant growth (Sun et al., 2023), and as one of the most vital biological processes in plants, it serves as a key indicator of vegetation response to environmental changes (Xu, 2016). Research indicates that heat stress adversely affects plant photosynthesis; however, non-extreme high temperatures may induce acquired thermotolerance in plants (Shi et al., 2023). Photosynthetic rate, stomatal conductance, and intercellular CO_2_ concentration interact closely, with stomatal conductance affecting the efficiency of CO_2_ uptake from the atmosphere, and intercellular CO_2_ concentration being a primary factor influencing photosynthetic rate variations (Chen et al., 2010; Zhang et al., 2023). Stomata are critical channels for plant life activities, dynamically regulating physiological balance in response to vegetation status changes (Shi and Leng, 1995; Wang, 2018). Although stomatal conductance tends to increase with rising temperatures, high heat can inhibit it (Gao et al., 2016). In this study (**Figure 5**), stomatal conductance in T was consistently lower than CK, showing a trend of initial increase followed by decrease. Reduced stomatal conductance led to lower intercellular CO_2_ concentration in T compared to CK, while photosynthetic rate initially increased but declined over time. The decline in photosynthetic capacity corresponded with a reduction in leaf chlorophyll content, resulting in delayed or slowed vegetation growth (Wahid et al., 2007), consistent with previous findings (Mu et al., 2014; Zhang et al., 2024). Chlorophyll fluorescence, an important indicator of photosynthesis, can be modulated under heat stress to mitigate photodamage (Guo et al., 2006; Xia et al., 2025). Wang et al. (2022) observed in a gradient temperature study that at 120 °C, Fv'/Fm' initially increased then decreased, while qP steadily declined, consistent with results in the present study (Wang et al., 2022).

The temporal variation trends of raw spectral reflectance were generally similar between T and CK, with T consistently exhibiting higher reflectance. Differences in the visible light range were limited to the budding stage, whereas significant differences in the near-infrared plateau persisted throughout, indicating that alfalfa’s near-infrared spectral reflectance is more sensitive to heat stress. Derivative transformations of raw spectra effectively refine spectral feature information (Zornoza et al., 2008). By constructing vegetation indices and triangular spectral parameters, combined with correlation analysis and the Successive Projections Algorithm (SPA), sensitive spectral features were identified alongside SPAD and photosynthetic parameter data. The study found that sensitive spectral bands for physiological indicators at different growth stages were mainly distributed in the infrared region, with first-derivative spectral vegetation indices showing stronger correlations, consistent with previous research (Guo et al., 2021; He et al., 2021; Luo et al., 2022). Regression analysis methods yielded satisfactory predictive performance (Liu et al., 2017). Among the three regression models—Support Vector Regression (SVR), Partial Least Squares Regression (PLSR), and Random Forest Regression (RFR)—SVR consistently achieved the best prediction accuracy across the three growth stages. The budding stage exhibited the highest predictive precision, with T’s SPAD predicted using FDNDVI (R² = 0.77, RMSE = 3.50). The spectral response correlation of photosynthetic parameters across growth stages aligned with SPAD results, with vegetation indices again providing the best model predictions; NDVI yielded optimal results (A: R² = 0.88, RMSE = 1.39; qN: R² = 0.68, RMSE = 0.07). The high accuracy of vegetation physiological parameter prediction models constructed using vegetation indices demonstrates the potential of spectral data in studying vegetation responses to heat stress. This approach can play an indispensable role in monitoring and early warning of spontaneous combustion risks on coal gangue dumps. Future integration with remote sensing imagery could enable assessment of vegetation heat stress intensity, identification of early potential combustion points, reduction of fieldwork, and minimization of reclamation losses in coal mining areas.

### 4.2 Mechanisms of heat stress effects on alfalfa physiological parameters and spectral responses

Spontaneous combustion of coal gangue dumps leads to increased soil temperature and alterations in soil physicochemical properties. Previous studies have shown that elevated soil temperature is a dominant factor affecting vegetation growth on coal gangue dumps (Ren et al., 2023). Soil temperature directly impacts plant roots, thereby influencing the transport of internal hormones within vegetation (Sun et al., 2023). Heat stress in the root zone had a stronger effect on vegetation compared with aerial heat stress, leading to more pronounced plant damage (Pramanik et al., 2018). Heat stress negatively affects vegetation growth (Wahid et al., 2007), with the severity depending on both the intensity and duration of the heat stress (Hüve et al., 2011), as well as the specific growth stage of the plants. High temperatures can inhibit seedling emergence, cause leaf wilting and senescence (Bahuguna and Jagadish, 2015), and may inflict irreversible damage to the photosynthetic apparatus (Mathur et al., 2014). Anatomically and morphologically, heat stress results in smaller plant cells, enhanced transpiration, water loss leading to stomatal closure, and reduced stomatal conductance (Gao et al., 2016), along with thickening of root xylem vessels (Bañon et al., 2004; Zhu et al., 2011). Plant functional morphology reflects the physiological state of vegetation, while physiological parameters serve as indicators of plant vitality (Ricotta et al., 2015). Vegetation spectral curves exhibit distinct “peaks” and “valleys,” with leaf reflectance at various wavelengths closely linked to cellular structure and physiological traits. Consequently, correlations between physiological parameters and spectral responses exist, enabling the widespread application of hyperspectral remote sensing technology in plant research (Elvidge, 1990; Kokaly et al., 2009; Violle et al., 2007).Under heat stress, photosynthesis weakens and intercellular CO_2_ concentration decreases. Heat stress significantly alters leaf reflectance spectral characteristics, with the shortwave red-edge region (680–750 nm) exhibiting “red-shift” or “blue-shift” phenomena, directly reflecting heat effects on the spectrum. In the experiment, heat stress also induced a reduction in leaf water content, which was manifested by an increase in reflectance within the shortwave infrared (SWIR) region, indicating that temperature exerted a regulatory effect on the leaf water status. Differences in correlation coefficients between various vegetation indices and spectral bands suggest that heat stress can be identified and quantified using specific spectral indices. The use of sensitive bands enhanced by first-derivative spectra or distinct triangular spectral parameters facilitates improved sensitivity in monitoring subtle physiological changes.

### 4.3 Limitations and future perspectives

This study investigated the spectral response patterns of physiological indicators in alfalfa under heat stress by measuring photosynthetic parameters and canopy spectral data. It aimed to clarify the optimal monitoring period and data processing models for alfalfa as the observation target. However, several limitations remain. The best observation period identified was the budding stage. Hyperspectral data were collected using a spectrometer, and morphological differences across alfalfa’s growth stages may have introduced variability in data accuracy. Additionally, the predictive models employed were empirical and not specifically optimized, which may have affected the precision of results. Improving accuracy will require validation with larger datasets. Moreover, spontaneous combustion of coal gangue dumps is a dynamic process, whereas this study utilized a constant temperature experiment, thus differing from the actual field conditions.

In summary, heat stress influences vegetation physiological activities and spectral characteristics. The coupling mechanism between physiological and spectral responses induced by heat stress not only deepens understanding of plant stress physiology but also provides theoretical support for remote sensing–based heat stress monitoring. Future research should focus on dynamic vegetation observation under variable temperature conditions to explore alfalfa’s spectral response characteristics and differences in heat stress responses. This will enhance the adaptability and accuracy of hyperspectral technology for monitoring spontaneous combustion in coal gangue dumps, offering valuable support for early warning systems.

## 5 Conclusions

This study used alfalfa as the research object. By conducting simulated high-temperature stress experiments representative of coal gangue dumps, SPAD, six photosynthetic parameters, and corresponding time-series spectral characteristics were obtained. Prediction models linking physiological indicators with spectral parameters were established to explore the response relationships between plant physiological traits and spectral features. The main conclusions are summarized as follows:

1. The SPAD values and photosynthetic parameters of alfalfa in both the CK and T groups showed similar temporal patterns across growth stages, characterized by an initial increase followed by a decline. However, heat stress significantly reduced the levels and variation amplitudes of these indicators in the T group. Among the photosynthetic parameters, gsw and qP responded most strongly during the branching and flowering stages, whereas A and qN were more sensitive during the budding stage.
2. Correlation analysis between physiological indicators and spectral features, including raw spectra, first-derivative spectra, and triangular parameters, indicated that FDNDVI and FDDVI exhibited the strongest responses under different treatments. First-derivative spectral features showed higher correlations with SPAD than raw spectra, while index-based spectral features were more strongly correlated with photosynthetic parameters.
3. Among the three predictive models (SVR, RFR, and PLSR), the SVR model achieved the best performance for both SPAD and photosynthetic parameter prediction, with optimal results obtained during the budding stage under the T group. Overall, FDNDVI and NDVI consistently yielded higher predictive accuracy, suggesting that spectral vegetation indices have the greatest potential for heat stress diagnosis during the budding stage.

Coal gangue spontaneous combustion is a dynamic process accompanied by temporal variations in soil temperature. As this study only considered constant-temperature scenarios, future research should integrate dynamic temperature simulations with field observations to improve the applicability and reliability of predictive models for coal gangue spontaneous combustion monitoring and early warning.

## Data Availability

The raw data supporting the conclusions of this article will be made available by the authors, without undue reservation.
